# Prevalence of Myopia and Its Risk Factors in Urban School Children in Delhi: The North India Myopia Study (NIM Study)

**DOI:** 10.1371/journal.pone.0117349

**Published:** 2015-02-26

**Authors:** Rohit Saxena, Praveen Vashist, Radhika Tandon, R. M. Pandey, Amit Bhardawaj, Vimala Menon, Kalaivani Mani

**Affiliations:** 1 Department of Ophthalmology, Dr Rajendra Prasad Center for Ophthalmic Sciences, All India Institute of Medical Sciences, New Delhi, India; 2 Department of Community Ophthalmology, Dr Rajendra Prasad Center for Ophthalmic Sciences, All India Institute of Medical Sciences, New Delhi, India; 3 Department of Biostatics, All India Institute of Medical Sciences, New Delhi, India; Wenzhou Medical University, CHINA

## Abstract

**Purpose:**

Assess prevalence of myopia and identify associated risk factors in urban school children.

**Methods:**

This was a cross-sectional study screening children for sub-normal vision and refractive errors in Delhi. Vision was tested by trained health workers using ETDRS charts. Risk factor questionnaire was filled for children with vision <6/9.5, wearing spectacles and for a subset (10%) of randomly selected children with normal vision. All children with vision <6/9.5 underwent cycloplegic refraction. The prevalence of myopia <-0.5 diopters was assessed. Association of risk factors and prevalence of myopia was analyzed for children with myopia and randomly selected non myopic children and adjusted odds ratio values for all risk factors were estimated.

**Results:**

A total number of 9884 children were screened with mean age of 11.6 + 2.2 years and 66.8% boys. Prevalence of myopia was 13.1% with only 320 children (24.7%) wearing appropriate spectacles. Mean myopic spherical error was -1.86 + 1.4 diopters. Prevalence of myopia was higher in private schools compared to government schools (p<0.001), in girls vs. boys (p = 0.004) and among older (> 11 years) children (p<0.001). There was a positive association of myopia with studying in private schools vs. government schools (p<0.001), positive family history (p< 0.001) and higher socio-economic status (p = 0.037). Positive association of presence of myopia was observed with children studying/reading > 5 hours per day (p < 0.001), watching television > 2 hours / day (p < 0.001) and with playing computer/video/mobile games (p < 0.001). An inverse association with outdoor activities/playing was observed with children playing > 2 hours in a day.

**Conclusion:**

Myopia is a major health problem in Indian school children. It is important to identify modifiable risk factors associated with its development and try to develop cost effective intervention strategies.

## Introduction

Myopia is a very common cause of visual impairment throughout the world [1,2]. Though, the prevalence of myopia varies by country, age and ethnic group, it is a major cause of visual impairment in both the developed and the developing world [2,3,4]. In East Asia the prevalence of myopia has been reported to be very high particularly in Japan [5], South Korea [6,7], Singapore [8], Taiwan [9,10], Hong Kong [11–12], and China [13–16] though much lower rates have been reported from South Asia and India[17–21]. While there are no large scale studies in India for assessing the magnitude of myopia in the school going population, available studies show higher prevalence rates in urban areas compared to rural areas [17–19]. Besides risk of ametropic amblyopia in growing children and the inconvenience and cost of spectacles or contact lenses, 10–20% have high myopia predisposing them to severe irreversible visual impairment [22].

As there is no well established or universally accepted method for the prevention of myopia onset, it is important to identify modifiable risk factors associated with its development and create cost effective interventional strategies. This study was undertaken with the aim to assess the magnitude of myopia in school going children in Delhi and identify the factors associated with it.

## Methods

A cross sectional study design was used in which children studying in classes 1 to 9 in different schools of Delhi were screened for sub-normal vision and prevalence of myopia.

Ethics statement: The study was approved by the institutional ethics committee of the All Indian Institute of Medical Sciences, New Delhi and followed the tenets of the Declaration of Helsinki for biomedical research. Permission for conducting the study in the selected schools was taken from the District Education Authority. A Participant Information Sheet explaining the study aims and objectives, the detailed procedure that would be carried out in the study and any adverse affects of dilatation along with a form to sign for providing the informed consent for the procedure was sent to all the parents. The forms were in English and the local vernacular language which is Hindi. This included permission to take vision, examine the eye, ask a questionnaire about demographic details and habits of the child and dilate the eye for refraction if required. Telephone numbers of the study investigators was provided in the Participant Information Sheet so that parents could ask if there were any doubts regarding the procedure. In all cases where the consent form was not returned or the parents had any doubts regarding the procedure, they were contacted by telephone and all questions regarding the study were answered. If the parents did not return the signed informed consent form, the child was not enrolled in the study. All examinations were carried out in the presence of an appointed representative of the school principal.

Delhi has nine districts of which two (South and West) were randomly selected. A list of all the registered schools in these two districts of Delhi was obtained and 10 schools were randomly selected from each district. All the districts have population of all socio economic strata therefore the children covered should be representative of the population of Delhi. Permission for conducting the study in the selected schools was taken from the District Education Authority. Two distinct types of schools were listed: Municipal Corporation / Delhi Administration/Government aided schools and privately funded schools. This distinction between schools was made as there is a difference in the socio-economic status (SES) of the children attending these schools. The private schools have higher fees so usually children of high and middle income families are enrolled in them compared to government schools with very low fees structure and therefore the enrolled children belong to the lower SES.

Examination was done during school hours and all the children studying in classes I–IX in the school were enrolled. The study was preceded by one month of staff training and field exercise in a school not a part of the study. The data collection instrument was a structured questionnaire and was pre-tested in 2 schools that were not a part of the study. The pilot study included evaluation of inter-observer agreement among field workers for vision assessment and filling of the questionnaire. The questions were asked in local vernacular language that is Hindi and is understood by all the children and parents and the answers were recorded in English. The questionnaire was filled by asking the details from the child and by telephonic interview of one or both parents and was aimed to determine the genetic and environmental factors affecting the development of myopia in these children. The details regarding the time spent on various activities was asked individually for every day of the week i.e. Monday to Sunday and the total number of hours engaged in each activity was recorded as hours spent for each activity per week. Time spent in each activity was asked separately for home and school. These details were cross checked by confirming them with the teacher and by telephonic interviews with the parents.

The vision of the child was recorded as the smallest line read with one or no errors using the “E” letters of the Early Treatment Diabetic Retinopathy Study (ETDRS) vision chart under ambient room lighting by a health worker especially trained for the study. Presenting visual acuity was tested in the right eye first followed by the left eye. All those children unable to read the 6/9.5 letters on examination by the health worker or those wearing spectacles, a risk factor Questionnaire was filled. The questionnaire was also filled for 10% of those children who had normal vision on testing by field worker (non myopic group). These children were selected randomly among all the children having normal vision as identified by the trained health worker.

Anthropometric measurements were done for all the children in whom the risk factor questionnaire was filled. Weight was taken using a regularly calibrated scale measuring weight in kilograms up to100 grams (0.1 kg) units and the height using a stadiometer with 1 mm unit.

All those children unable to read the 6/9.5 letters or those previously wearing spectacles were referred to an ophthalmic technician for refraction. Refraction was done in 2 stages, first under cycloplegia using eye drops 2% homatropine which was instilled in the inferior conjunctival cul-de-sac twice at an interval of ten minutes. If after 20 minutes the pupillary light reflex was still present, a third drop was administered. Cycloplegia was considered complete if pupil dilated to 6 mm or more and there was no pupillary reflex. Retinoscopy was done using a streak retinoscope and a hand held autorefractometer (Retinomax K-Plus; Nikon Corp., Tokyo, Japan). The autorefractometer was calibrated at the beginning of each working day. Subjective acceptance was done at a following visit after a week and post mydriatic acceptance was checked. The refractive error was documented based on the subjective acceptance. All children prescribed with spectacles were provided spectacles at concessional rate. All children who were unable to read the 6/12 letters on the ETDRS charts even after refraction or children with other ocular illnesses underwent a complete ophthalmic examination by an ophthalmologist and were further managed gratis at a tertiary care facility.

The spherical equivalent (SE) was calculated as the numerical sum of the sphere and half the cylinder. Myopia was defined as spherical equivalent refractive error of -0.50 D or worse in either or both eyes and all myopic children were included for statistical analysis.

The sample size was estimated using the following formulae: 2 Z² P Q W/ L²

The Assumed Population Prevalence (P) = 7.5% in the age group of 5 to 15 years [18] Q = 100-P = 92.5%, Z = 1.96 with L = maximum acceptable random sampling error = 10% of prevalence. W = Design effect = 2, Sample Size was estimated as 10,000 children.

The statistical analysis was carried out using STATA software 13.0 (College Station, USA). The data was presented as number (%) or mean ± SD as appropriate. The magnitude of myopia was presented as prevalence (95% Confidence Interval-CI). The risk factors were divided into demographic (non modifiable) and behavioral (modifiable) risk factors. Logistic regression analysis was carried out to find the risk factors of myopia. The results were reported as Odds Ratio (95% CI). The p value < 0.05 was considered statistically significant.

## Results

This was a cross sectional study with the aim of enrolling children studying in different schools of Delhi. The total number of children enrolled in the 20 selected schools was 10,114 and 9884 children (97.7% coverage) were screened in the study. There were 11 government and 9 private schools. The aim was to have a relatively equal number of children from both the type of schools. The number of children examined in government schools was 4215 with 97% coverage of the enrolled children and 5669 children in private schools with coverage of 97.7% of the total enrolled children in the selected classes. The mean age of children examined in the study was 11.6 ± 2.2 years (range of 5–15 years) with 6602 (66.8%) boys. The proportion of boys and girls in the enrolled and examined children was similar. The age and gender distribution of the enrolled and examined children is given in Table 1.

**Table 1:** Age and gender wise distribution of enrolled and examined children.

Age in years	Total enrolled		Total (%)	Total examined		Total (%)
	Boys (%)	Girls (%)		Boys (%)	Girls (%)	
5–10	1969 (61.2)	1247 (38.8)	3216 (100.0)	1942 (61.4)	1221 (38.6)	3163 (100.0)
11–13	3183 (66.7)	1589 (33.3)	4772 (100.0)	3100 (66.7)	1551 (33.3)	4651 (100.0)
14–15	1600 (75.3)	526 (24.7)	2126 (100.0)	1560 (75.4)	510 (24.6)	2070 (100.0)
**Total**	**6752 (66.8)**	**3362 (33.2)**	**10114(100.0)**	**6602 (66.8)**	**3282 (33.2)**	**9884 (100.0)**

Of the 9884 children examined in the study 572 (5.8%) had presenting vision <6/12 in the better eye; 455 of these (79.5%) were myopic. Mild visual impairment (presenting vision < 6/12–6/19 in the better eye) was present in 322 (3.3%) with 249 (77.3%) were myopes. Table 2. There were 1073 (10.8%) children with presenting vision ≤6/12 in the better eye with 818 (76.2%) of these were due to myopia. Of all the children screened 14.5% were referred for refraction (VA <6/9.5 any eye) and the prevalence of myopia in these children was 13.1% (0–95% CI = 12.5, 13.8). Mean myopic spherical error was-1.86 ± 1.4 diopters (range: -10.25 to-0.5). Low myopia (≥ -3 diopters) was found in 1120 children (86.3%) and only 20 children (1.5%) had high (≤ -6 diopters) myopia. The mean age of children with myopia (n = 1297) was 12.1 ± 1.9 and those without myopia (n = 8587) was 11.5 ± 2.2. Of these 428 children (33%) were already wearing spectacles though 108 children (8.3%) were unable to read 6/9.5 with their current spectacles. Therefore the total unmet need of myopia was 75.3% (977/1297). In private schools 27.8% (267/963) myopic children were wearing spectacles compared to 15.9% (53/334) in government schools (p<0.001).

**Table 2:** Presenting visual acuity of better eye for all children and those diagnosed with myopia.

Vision category	Myopic children n(%)	All examined children n(%)
**Normal(6/6–6/12)**	842 (64.9)	9312 (94.2)
**Mild visual impairment (<6/12–6/19)**	249 (19.2)	322 (3.3)
**Moderate visual impairment (<6/19–6/60)**	204 (15.7)	247 (2.5)
**Severe visual impairment—blind (<6/60)**	2 (0.2)	3 (0.0)
**Total**	**1297 (100%)**	**9884 (100%)**

The prevalence of myopia was higher among private schools (17%) compared to government schools (7.9%) (p<0.001), among girls 14.5% (476/3282) compared to boys 12.4% (821/6602) (p = 0.004) and among older children (≥ 11 years of age) (p<0.001). (Table 3).

**Table 3:** Association of myopia with age, gender and type of school.

Category	Myopia (%)n = 1297	Total n = 9884	Prevalence (95% CI)	p-value
**Age in years**				
**5–10**	267 (20.7)	3163(32.0)	8.4 (7.5, 9.4)	**<0.001**
**11–13**	713 (55.0)	4651(47.1)	15.3 (14.3, 16.4)	
**14–15**	317 (24.3)	2070 (20.9)	15.3 (13.8, 16.9)	
**Gender**				
**Boys**	821 (66.8)	6602 (66.8)	12.4 (11.6, 13.2)	**0.004**
**Girls**	476(33.2)	3282 (33.2)	14.5 (13.2, 15.7)	
**Type of School**				
**Private School**	963 (74.2)	5669 (57.4)	17.0 (16, 17.9)	**<0.001**
**Government School**	334 (25.8)	4215 (42.6)	7.9 (7.1, 8.73)	

**Fig 1:**
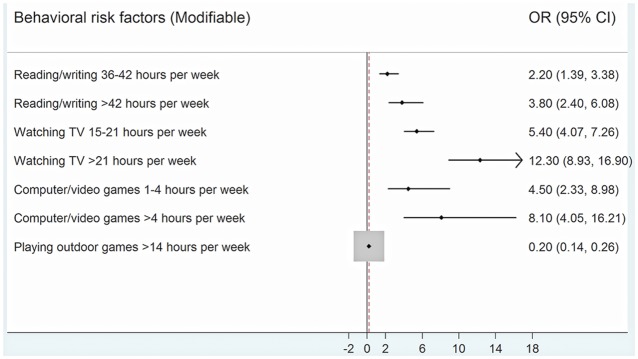
Forest plot showing the odds ratio (95% confidence interval) for each behavioral risk factor adjusted for other behavioral risk factors and demographic variables (age, gender, type of school, family history of glasses, mother’s education and socio-economic status).

The demographic and behavioral (modifiable) risk factors were analyzed for both children with myopia (n = 1297) and the randomly selected non myopic children (n = 1153) and adjusted odds ratio values for all risk factors were estimated. Each risk factor was adjusted for the other risk factors given in Table 4. The results showed that there was a positive association of myopia with those studying in private schools compared to government schools, positive family history (parents, siblings) of wearing spectacles (p< 0.001 for both) and higher socio-economic status (p = 0.037). Age or gender of the child and educational attainment for the mother was not associated with an increased risk of myopia in the child. On examining the behavioral risk factors (Fig. 1.), a positive association of presence of myopia was observed with children studying/reading > 5 hours in day (p < 0.001), watching television > 2 hours / day (p < 0.001) and with playing computer/video/mobile games (p < 0.001). An inverse association with outdoor activities/playing was observed with children playing > 2 hours in a day (observed in only 5% child with myopia compared to 47.4% in non myopic children; p < 0.001). (Table 4).

**Table 4:** Demographic and Behavioral risk factors of myopia: Results of binary and multi variable logistic regression analysis.

Risk factors	Myopic (%) n = 1297	Non-Myopic (%) n = 1153	Unadjusted OR (95% CI)	p- value	Adjusted OR (95% CI)	p-value
**Age in years**						
**5–10**	267 (20.61)	369 (32.0)	1.0		1.0	
**11–13**	713 (55.0)	556 (48.2)	1.8 (1.46–2.14)	<0.001	---	0.913
**14–15**	317 (24.49)	228 (19.8)	1.9 (1.52–2.42)	<0.001	---	0.216
**Gender**						
**Boy**	821(63.3)	767(66.5)	1.0		1.0	
**Girl**	476(36.7)	386(33.5)	1.2 (0.97–1.36)	0.096	1.2 (0.96–1.36)	0.139
**School**						
**Government School**	334(25.7)	541(46.9)	1.0		1.0	
**Private School**	963(74.3)	612(53.1)	2.5 (2.15–3.02)	<0.001	3.6 (2.77–4.73)	**<0.001**
**Any Family Members Wearing Spectacles**						
**No**	613(47.3)	974(84.5)	1.0		1.0	
**Yes**	684(52.7)	179(15.5)	6.1 (5.00–7.36)	<0.001	3.4 (2.63–4.35)	**<0.001**
**Mother’s Education**						
**Illiterate-Primary School**	441 (34.2)	450 (39.2)	1.0		1.0	
**Middle-Post High School**	652 (50.5)	576 (50.2)	1.2 (0.97–1.37)	0.102	--------	
**Graduate & above**	197 (15.3)	122 (10.6)	1.6 (1.26–2.13)	<0.001	1.4 (0.96–1.95)	0.082
S**ocioeconomic status**						
**Lower—Upper lower**	141 (11.2)	196 (17.6)	1.0		1.0	
**Lower Middle**	418 (33.0)	387 (34.8)	1.5 (1.16–1.94)	0.002		0.410
**Upper Middle—Upper**	706 (55.8)	529 (47.6)	1.8 (1.45–2.36)	<0.001	1.4 (1.08–1.72)	**0.037**
**No. of hours per week of reading/writing at school and home**						
**28–35**	52 (4.0%)	200 (17.3)	1.0		1.0	
**36–42**	670 (51.7)	698 (60.5)	3.7 (2.67–5.09)	<0.001	2.2 (1.39–3.38)	**<0.001**
**>42**	575 (44.3)	255 (22.1)	8.7 (6.18–12.17)	<0.001	3.8 (2.40–6.08)	**<0.001**
**No. of hours per week of watching television**						
**0–14**	133 (10.2)	682 (59.1)	1.0		1.0	
**15–21**	512 (39.5)	312 (27.1)	8.4(6.66–10.62)	<0.001	5.4 (4.07–7.26)	**<0.001**
**>21**	652 (50.3)	159 (13.8)	21.0(16.30–27.11)	<0.001	12.3 (8.93–16.90)	**<0.001**
**No. of hours per week of using computers and video games**						
**0**	14 (1.1)	172 (14.9)	1.0		1.0	
**1–4**	660 (50.9)	723 (62.7)	11.2(6.43–19.53)	<0.001	4.5 (2.33–8.98)	**<0.001**
**>4**	623 (48.0)	258 (22.4)	29.7(16.88–52.13)	<0.001	8.1 (4.05–16.2)	**<0.001**
**No. of hours per week of playing outdoor games**						
**0–14**	1232 (95.0)	606 (52.6)	1.0		1.0	
**>14**	65 (5.0)	547 (47.4)	0.1 (0.04–0.07)	<0.001	0.2 (0.14–0.26)	**<0.001**

The distribution of the modifiable risk factors was compared according to gender and type of school to identify the cause of higher prevalence of myopia among girls and in private schools. Table 5. The results showed that children in private schools spent a significantly greater number of hours in reading/ writing at home and on playing computer and video games (p<0.001 for both). Children in government schools also spent a significantly greater number of hours playing outdoors (p< 0.001). Girls also spent a greater number of hours in reading/ writing at home compared to boys (p<0.01) while boys spent greater number of hours playing computers and video games but also spend a greater number of hours in outdoor games (p<0.001 for both).

**Table 5:** Distribution of modifiable risk factors according to gender and type of school.

Risk Factors (Mean hours per week)	Boys (n = 821)	Girls (n = 476)	P value	Government Schools (n = 334)	Private Schools (n = 963)	P value
**Reading in school**	28.3 ± 1.1	28.4 ± 1.3	0.2	28.1 ± 1.1	28.8 ± 1.2	0.09
**Reading at home**	14.1 ± 3.7	14.8 ± 3.6	**<0.01**	13.7 ± 3.6	14.9 ± 3.7	**<0.001**
**Watching TV**	22.5 ± 5.6	23.0 ± 6.1	0.13	22.8 + 5.7	21.9 + 5.7	0.08
**Playing computers & video games**	5.6 ± 3.5	4.3 ± 2.8	**<0.001**	4.5 ± 3.3	5.3 ± 3.4	**<0.001**
**Playing outdoors**	12.9 ± 2.1	11.7 ± 2.12	**<0.0001**	12.8 ± 3.4	12.3 ± 2.1	**0.0002**

## Discussion

The aim of the current study was to evaluate the prevalence of myopia and the possible risk factors causing it in Indian school going children. Our study covered 97.7% of the 10114 children enrolled in the selected schools so the results should be reflective of the pattern expected in urban schools in India. The proportion of boys in the enrolled and examined children was 66.8%. On evaluating the age and gender distribution, there was a progressive reduction in the number of girls among older children. As the gender ratio was similar in the enrolled and the examined children, the reduced number of girls observed in the study is truly reflective of the state of enrolment of girls and is not due to greater absenteeism among girls. This could be due to a possible drop out in the number of girls as they get older and is reflective of the low emphasis our society places on education of the girl child.

Number of children with presenting vision <6/12 in the better eye were 572 (5.8%) with 455(79.5%) of these were due to myopia. Therefore not only is visual impairment in school children significant, a large proportion of the visual impairment can be eliminated by provision of appropriate spectacles. In our study 10.8% children had presenting vision ≤ 6/12 in the better eye with nearly 80% due to myopia. Surveys in urban and rural India [17,18] Nepal [20] and in China [23] have reported ≤ 6/12 in the better eye ranging from 56%–89.5% with almost 80% achieving normal vision if all children were provided with appropriate spectacles. They have recommended that as a large percentage of children with vision-reducing refractive error are apparently not wearing spectacles, population-based screening programs may be necessary to reduce visual impairment among school-aged children [18]. Though population based screening for childhood refractive errors may not be feasible in India, the school eye screening programme should be strengthened to improve coverage and ensure annual screening of children and provision of subsidized spectacles. Substantial benefit could be realized by the provision of refraction services and the availability of affordable spectacles.

The results showed that 14.5% children were referred for refraction (VA <6/9.5 any eye) and of these the prevalence of myopia in school going children was 13.1% with a mean myopic spherical error of-1.86 ± 1.4 diopters. Though the prevalence of myopia is low compared to those reported from other countries in East Asia [5–16], it is higher than those previously reported by studies from India [17–19] and Nepal [20]. Due to different refractive error cut offs, different sample population and different methodologies of the previous Indian studies, it is difficult to state whether this difference indicates an actual increase in the prevalence but 13.1% prevalence in children in a country of 1.2 billion with over 20% in 5–15 years age-group, implies that myopia is a significant public health problem in India. Moreover Indians who have immigrated to regions with high prevalence of myopia have shown significantly higher myopia [24] compared to the prevalence of myopia reported from India. Thus there is little evidence supporting an intrinsically higher prevalence of myopia, or a greater susceptibility to environmental risk factors in populations of East Asian origin compared to those from South Asia or the West.

The unmet need to correct refractive errors in children is significant with over 75% children in urban schools not wearing spectacles for their myopia or were using under-corrected spectacles /wrong prescriptions. In private schools a significantly higher number of children with myopia were wearing spectacles compared to government schools showing greater coverage in these schools. This could possibly also be due to the better awareness, pressure for performance and results and greater health seeking behavior. However numerous barriers to the use of spectacles among these children still exist [25,26] and counseling and motivation is necessary to improve compliance.

Our study found a higher prevalence of myopia among children studying in private schools compared to government schools. Although overall academic curriculum and the hours spent in school was similar between the two types of schools, children in private schools spent a significantly greater number of hours in reading and writing at home reflecting greater educational pressures and greater likelihood of attending extra classes and private tuitions. As education in India becomes more competitive and intensive, with greater emphasis on extra classes and private tuitions, the magnitude of myopia in Indian children is likely to increase. Also, as these children are from higher socio-economic status (SES) they are more likely to have access to computer, video games and television, a significantly greater number of hours were spent in playing computers and video games compared to children from government schools.

Higher prevalence of myopia among girls as is seen in our study has been reported before. Our study observed that girls spent greater number of hours in reading and writing at home compared to boys and significantly lesser hours outdoors. This increase in time spent in reading and reduced outdoor activity predisposes them to development of myopia. Therefore girls constitute a high risk group and special efforts should be made to examine girls in this age group and also encourage them to play outdoors.

We found that myopia was more prevalent in children with positive family history of myopia and higher socio-economic status. The impact of genetic factors in the prevalence of myopia has been extensively reported and sibling risk ratios are generally high. However families share the same environment, nutrition, culture and education besides the genetic makeup making it difficult to conclusively prove the influence of genes in the development of myopia^1^. Therefore more than the genetic factors the shared social and environmental influences are more likely to promote the development of myopia.

Our study has evaluated the effect of behavioral (modifiable) risk factors on myopia. The results show that near related activity such as study/reading > 5 hours in day, watching television > 2 hours / day and playing computer/video/mobile games increased the risk of developing myopia. We also found that outdoor activities/playing protected from myopia and the prevalence of myopia was significantly less in children who were playing > 2 hours in a day. Numerous studies have reported a strong environmental effect on prevalence of myopia in a population and a strong association with higher grades at school, accelerated learning streams and international educational performance has been reported [27–29]. A Longitudinal study suggests that the chance of becoming myopic is significantly reduced if time spent outdoors is increased from a mean of 7.98 hours a week to 11.65 hours per week (p< 0.001) a figure similar to our study [30]. Although it has not been directly linked to any particular sport or activity, increased amount of time spent outdoors in sunlight has been shown to reduce myopia prevalence rates possibly due to release of dopamine from the retina on exposure to light [31–32]. In our study more hours of studying and outdoor activity were independent risk factors and one was not due to absence of other factor.

The present study had some notable limitations. First, the data was based on findings from schools of one city (Delhi) hence the results may not be universally reflective especially due to the known variation reported from urban and rural regions [17–18]. The reliability of a questionnaire based system of collecting information can be questioned and though a multifactoral analysis was performed, there may be an overlap of the effect of commonly associated risk factors. Keeping these in view while interpreting the results one can still reliably conclude that myopia in school children in India is an important health issue associated with many lifestyle related modifiable risk factors which suggest that an increase in outdoor activity may help to reduce the magnitude of the problem.
